# Identification and Characterization of MicroRNAs in *Ginkgo biloba* var. *epiphylla* Mak

**DOI:** 10.1371/journal.pone.0127184

**Published:** 2015-05-15

**Authors:** Qian Zhang, Jihong Li, Yalin Sang, Shiyan Xing, Qikui Wu, Xiaojing Liu

**Affiliations:** Key Laboratory of Tree Germplasm Resources Research, College of Forestry, Shandong Agricultural University, Tai’an, Shandong, China; Kunming University of Science and Technology, CHINA

## Abstract

*Ginkgo biloba*, a dioecious plant known as a living fossil, is an ancient gymnosperm that stands distinct from other gymnosperms and angiosperms. *Ginkgo biloba* var. *epiphylla* (*G*. *biloba* var. *epiphylla*), with ovules borne on the leaf blade, is an unusual germplasm derived from *G*. *biloba*. MicroRNAs (miRNAs) are post-transcriptional gene regulators that play critical roles in diverse biological and metabolic processes. Currently, little is known about the miRNAs involved in the key stage of partly epiphyllous ovule germination in *G*. *biloba* var. *epiphylla*. Two small RNA libraries constructed from epiphyllous ovule leaves and normal leaves of *G*. *biloba* var. *epiphylla* were sequenced on an Illumina/Solexa platform. A total of 82 miRNA sequences belonging to 23 families and 53 putative novel miRNAs were identified in the two libraries. Differential expression analysis showed that 25 conserved and 21 novel miRNAs were differentially expressed between epiphyllous ovule leaves and normal leaves. The expression patterns of partially differentially expressed miRNAs and the transcript levels of their predicted target genes were validated by quantitative real time RT-PCR. All the expression profiles of the 21 selected miRNAs were similar to those detected by Solexa deep sequencing. Additionally, the transcript levels of almost all the putative target genes of 9 selected miRNAs were opposite to those of the corresponding miRNAs. The putative target genes of the differentially expressed miRNAs were annotated with Gene Ontology terms related to reproductive process, metabolic process and responding to stimulus. This work presents a broad range of small RNA transcriptome data obtained from epiphyllous ovule and normal leaves of *G*. *biloba* var. *epiphylla*, which may provide insights into the miRNA-mediated regulation in the epiphyllous ovule germination process.

## Introduction

MicroRNAs (miRNAs) are endogenous 18–25nt small non-coding RNAs that negatively regulate gene expression in eukaryotic species [1, 2]. In plant, miRNA genes are initially transcribed by RNA polymerase II to form primary miRNAs (pri-miRNAs). The pri-miRNAs are sequentially cleavage by DICER-Like1 to produce hairpin-like miRNA precursors (pre-miRNAs), which are then further cleaved to form their mature miRNA: miRNA* duplexes [3]. The mature miRNAs are incorporated into RNA-induced silencing complexes (RISCs) which induce cleavage and translational inhibiton of their target mRNAs [4]. After the initial discovery of plant miRNAs in 2002 [5], considerable numbers of miRNAs have been identified. Currently, 7385 mature miRNA sequences from 72 plant species have been deposited in miRBase (release 20.0, July 2013) [6]. However large numbers of these miRNAs are from model species for which the gemomes have been sequenced, including *Arabidopsis thaliana* [7], *Zea mays* [8], *Oryza sativa* [9] and *Populus trichocarpa* [10] and only a few miRNAs have been reported in gymnosperms [11–14]. There is very little information available on *Ginkgo biloba* miRNAs.

*G*. *biloba* is one of the most ancient living seed plants and, such as, it occupies an important position in the plant kingdom [15]. Usually, the ovuliferous structures develop at the axil of a leaf or a scale of the short shoot. Ovuliferous structures consist of a stalk with two sessile ovules on the top [16]. In 1891, abnormal ovulate organs were discovered in *G*. *biloba* by  Shirai in Yamanashi Prefecture, Japan [17, 18]. The peculiar pattern of these abnormal ovuliferous structure was the partly epiphyllous ovules, that developed on the leaf blades [17, 19]. In 1927, Makino named the mutant ‘*Ginkgo biloba* L. var. *epiphylla* Mak’(*G*. *biloba* var. *epiphylla*). The grafted *G*. *biloba* var. *epiphylla* shared similar growth characteristics with the parent tree, indicating the genetic stability of this mutant plant. It is well known that miRNAs play crucial roles in plant organ development [3, 4, 20], including somatic embryogenesis [21], female gamete formation [22] and ovule development [23]. There are fundamental differences between gymnosperms and angiosperms in ovule development and seed formation [24]. However, as with many other gene regulatory systems, miRNAs are conserved between angiosperms and gymnosperms, implying that, as in angiosperms, gymnosperm miRNAs may also be associated with differentiation and ovule development processes.

In this study, next-generation high-throughput sequencing technology was employed to sequence and identify *G*. *biloba* var. *epiphylla* miRNAs that may be involved in key stages of epiphyllous ovule germination. We obtained two small RNA libraries from epiphyllous ovule leaves and normal leaves. A total of 82 conserved miRNA sequences belonging to 23 miRNA families and 53 novel miRNA candidates were identified. The expression levels of selected miRNAs and the predicted target genes were validated by quantitative real time RT-PCR (RT-qPCR). Annotation of the target genes revealed their involvement in ovule processes, such as transcription regulation, substance/energy metabolism, and signal transduction.

## Results

### High-throughput sequencing of small RNAs from *G*. *biloba* var. *epiphylla*

**Fig 1:**
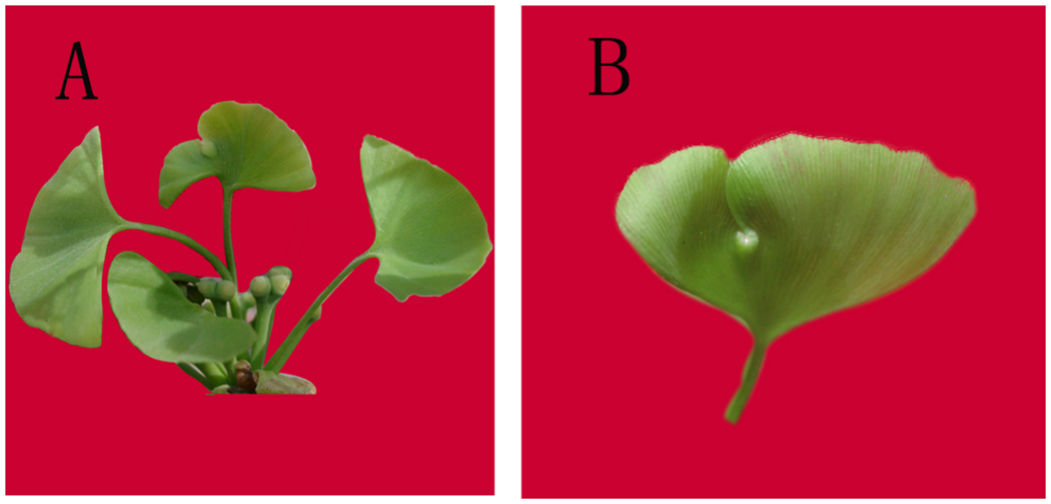
Epiphyllous ovule leaves and normal leaves from *G*. *biloba* var. *epiphylla*. (A) Abnormal ovuliferous structure with partly epiphyllous ovules leaves and normal leaves in the early stage of partly epiphyllous ovule germination (in mid-April). (B) Close-up of the epiphyllous ovules leaves (in mid-April).

Two small RNA (sRNA) libraries were constructed from epiphyllous ovule leaves (EL) and normal leaves (NL) and sequenced using the Solexa/Illumina sequencing platform (Fig 1). The deep sequencing yielded 22,401,208 and 19,042,060 raw reads from the EL and NL libraries, respectively. After removing adapters, contaminants, inserts, and low quality sequences, 21,717,220 (EL) and 18,380,325 (NL) clean reads, with lengths ranging from 18 to 30 nt, were obtained. Among the clean reads, 2,585,993 (EL) and 2,640,731 (NL) unique sequences were obtained (Table 1). In total, 88.49% of the clean sRNA reads, representing 15.91% of the unique sRNA sequences, were found in both libraries (Table 2). Of the unique sRNA sequences, 41.44% were EL-specific and 42.65% were NL-specific, suggesting that different gene expression regulation processes may exist between EL and NL.

**Table 1:** Summary of small RNA sequencing statistics.

Type	EL Count	NL Count
Total reads	22401208	19042060
High quality	21963192	18555840
Adaptor 3'-null	25591	40106
Insert-null	3804	6924
Adaptor 5'-contaminants	57159	87824
Sequences <18nt	158468	40202
PolyA	950	459
Clean reads	21717220	18380325

EL: Epiphyllous ovule leaves, NL: Normal leaves.

**Table 2:** Summary of the clean small RNAs in the epiphyllous ovule leaves and normal leaves libraries.

Category	Unique sRNA reads	Percent (%)	Total sRNA reads	Percent (%)
Total sRNAs	4509157	100.00	40097545	100.00
EL & NL	717567	15.91	35482562	88.49
EL-Specific	1868426	41.44	2252922	5.62
NL-Specific	1923164	42.65	2362061	5.89

EL: Epiphyllous ovule leaves, NL: Normal leaves.

**Fig 2:**
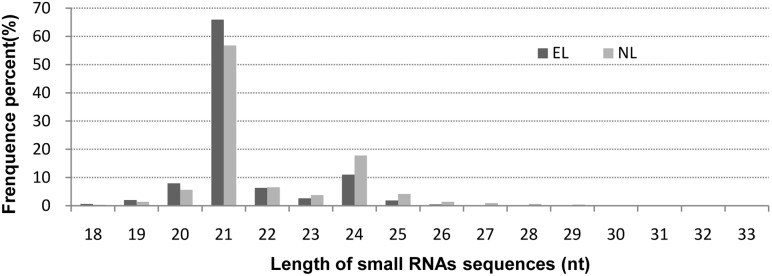
Length distribution of small RNAs from the epiphyllous ovule leaves (EL) and normal leaves (NL).

The unique sRNA sequences were mapped to the assembled unigene sequences in the *G*. *biloba* mRNA transcriptome database (41,525 unigene sequences with an average length of 961bp) because very few *G*. *biloba* genomic sequences were available. A total of 411,194 (15.9%) and 437,233 (16.6%) unique reads from the EL and NL libraries respectively, shared 100% identity with the *G*. *biloba* mRNA transcriptome sequences. After further removal of other non-coding sRNAs (rRNA, tRNA, snRNA, snoRNA) by BLAST searching against the Rfam database (http://rfam.sanger.ac.uk), 2,981,348 (EL) and 2,289,081 (NL) unique sequences were identified as candidate miRNAs. More than 50% of the sequences in the two libraries were not annotated after this search (Table 3). The length distributions of the sRNAs in both libraries revealed a major peak at 21nt and a minor peak at 24nt (Fig 2). This distribution pattern was similar to the distribution patterns in other gymnosperm species, such as *Taxus chinensis* [25], *Taxus mairei* [26] and *Pinus contorta* [27, 28] Although the distribution patterns in most angiosperms also showed two peaks at the same two lengths, the numbers of 24 nt reads were higher than the numbers of 21 nt reads, for example, in *Arabidopsis*[29], rice [30], *Liriodendron chinense* [31], amur grape [32], *Citrus trifoliata* [33], peanuts [34], and peach [35].

**Table 3:** Non-coding RNAs among the small RNAs in the epiphyllous ovule leaves and normal leaves libraries.

Category	Unique sRNA reads		Total sRNA reads	
	EL	NL	EL	NL
miRNA	20181(0.78)	16372 (0.62%)	3017210 (13.89%)	2384638 (12.97%)
rRNA	69876 (2.70%)	100345(3.80%)	801515 (3.69%)	1324626(7.21%)
snRNA	980 (0.04%)	1046 (0.04%)	7861 (0.04%)	13289 (0.07%)
snoRNA	462 (0.02%)	513 (0.02%)	1478 (0.01%)	1953 (0.01%)
tRNA	7818 (0.30%)	9278 (0.35%)	233893 (1.08%)	158418 (0.86%)
others	2486676 (96.16%)	251317(95.17%)	17655263(81.30%)	14497401(78.87%)
Total	2585993	2640731	21717220	18380325

EL: Epiphyllous ovule leaves, NL: Normal leaves.

### Identification of conserved miRNAs in *G*. *biloba* var. *epiphylla*

To identify conserved miRNAs in *G*. *biloba* var. *epiphylla*, we conducted BLASTN searches against the currently known mature plant miRNAs in miRBase 20.0 [6]. After removing low-abundance miRNAs (the sequences with read less than 10 reads), we identified a total of 82 conserved miRNA sequences belonging to 23 miRNA families shared 100% identity with known mature miRNA sequences from the two sRNA datasets, (S1 Table). All the miRNA sequences were identified in both datasets. The precursor sequences for the *G*. *biloba* var. *epiphylla* miRNAs were identified based on the *G*. *biloba* transcriptome-assembled sequences (S1 Fig).

**Fig 3:**
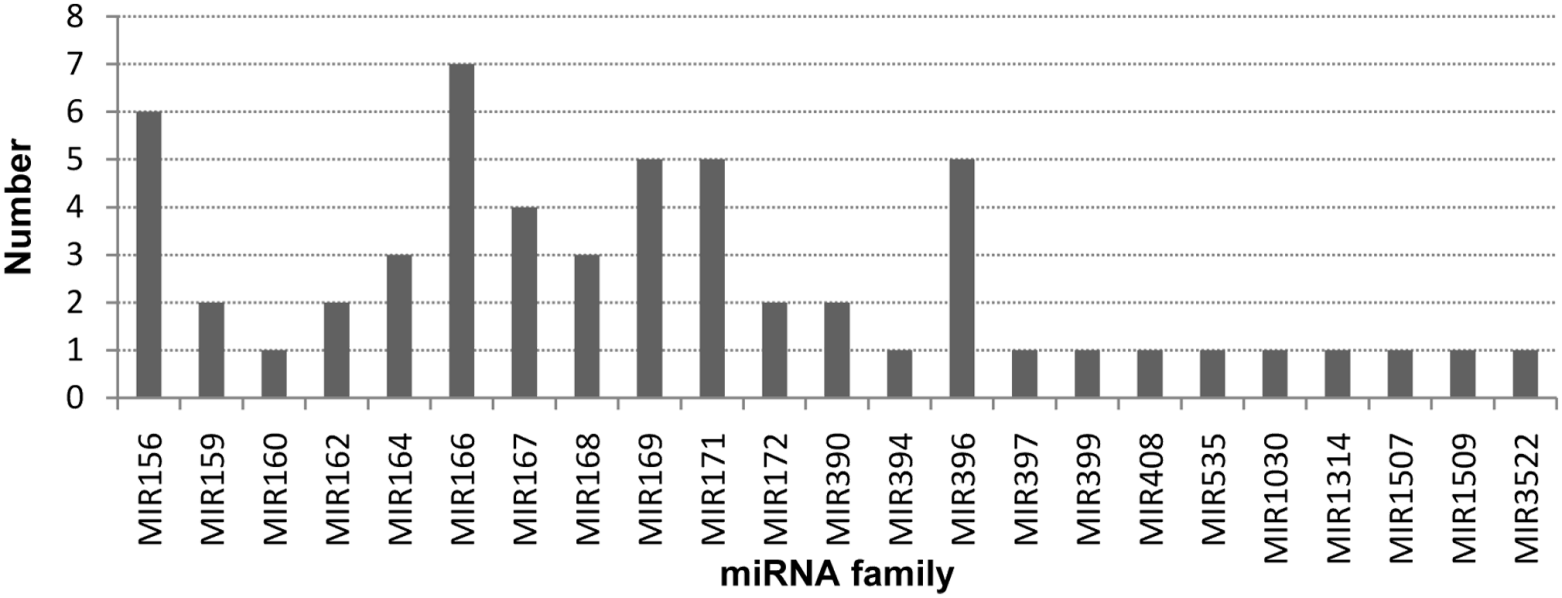
Numbers of members identified in the 23 conserved miRNA families.

Owing to the variability of the cleavage sites of DCL or subsequent processing, many miRNA genes give rise to multiple variants, which were annotated as miRNA isoforms [27]. Thus, different sequences that showed no more than two nucleotides in length differences in the 5’ or 3’ end in our dataset were combined and considered as one miRNA members. Overall, the 82 miRNA sequences were classified into 57 members. The largest family was MIR166, which contained 7 members. Among them, miR166a contained 6, while miR166b contained 9 isoforms. The MIR156 (6 members) and MIR167, 171, 396 (5 members) were the second and third largest miRNA families, respectively. Seven of the miRNA families contained between two and four members, while 11 of the families contained one single member (Fig 3). The identified miRNA families are conserved in a variety of plant species and 16 of the 23 miRNA families were found in both angiosperms and gymnosperms. One of the miRNAs, miR1314, has been found previously only in *Pinus densata* and *Pinus taeda* [13, 27], with no homologs found in angiosperms. Other miRNA families, for example miR3522, miR1507, miR1509 were identified in only a few species and, so far, miR1030 has been found in only *Physcomitrella patens* [36]. The conserved miRNA families with more members had higher abundance of expression, while the miRNA families with only one member showed lower expression levels. The members of the miR166, miR156, miR171, miR168 and miR172 families accounted for the overwhelming majority of the total number of conserved miRNAs.

### Identification of novel miRNAs in *G*. *biloba* var. *epiphylla*

The hairpin structure of pre-miRNAs has been used to help predict novel miRNAs. To identify novel miRNA candidates in *G*. *biloba* var. *epiphylla*, the unannotated sRNA sequences were matched against the assembled unigene sequences in the *G*. *biloba* mRNA transcriptome database. Novel miRNA candidates were aligned against the protein and miRBase databases to discard possible coding sequences and known miRNAs. Candidates with more than 10 reads were considered as putative novel miRNAs.Fifty-three putative novel miRNAs, including 25 miRNA*, were detected using the MIREAP algorithm (http://sourceforge.net/projects/mireap/). The pre-miRNA sequences, secondary structures, and the locations of the mature miRNAs in the stem-loop structure are listed in S2 Table. The pre-miRNAs ranged from 67 to 268 bp in length, with an average of 116 bp. The average minimum free energy (MFE) value obtained for these precursors was −46.0 kcal/mol, which is similar to the MFE values reported for *Taxus* precursors (−46.9 kcal/mol) [26] and trifoliate orange (*Citrus trifoliata*) precursors (−52.41 kcal/mol) [37], but a little higher than in the MFE values for *Z*. *mays* (−61.15 kcal/mol) and *A*. *thaliana* (−57.0 kcal/mol) [8].

The size distribution of the novel candidate miRNAs ranged from 20 to 23 nt, with 21 nt being the most abundant (66.0%). The first nucleotide bias of these candidate miRNAs was the common 5' terminal uridine (U) nucleotide, in accordance with previous findings [8]. The read counts for these miRNAs in the two datasets were low. The most abundant novel candidate miRNA was gbi-miRN17 with 94,497 and 75,098 reads in the EL and NL datasets respectively. In general, the read counts of most of the miRNA*s were much lower than the corresponding miRNAs. Although the identified novel miRNAs and miRNA*s displayed much lower expression levels than the conserved miRNAs, they may play a specific role during the development stage in specific tissues.

### Expression profile of conserved and novel miRNAs

**Fig 4:**
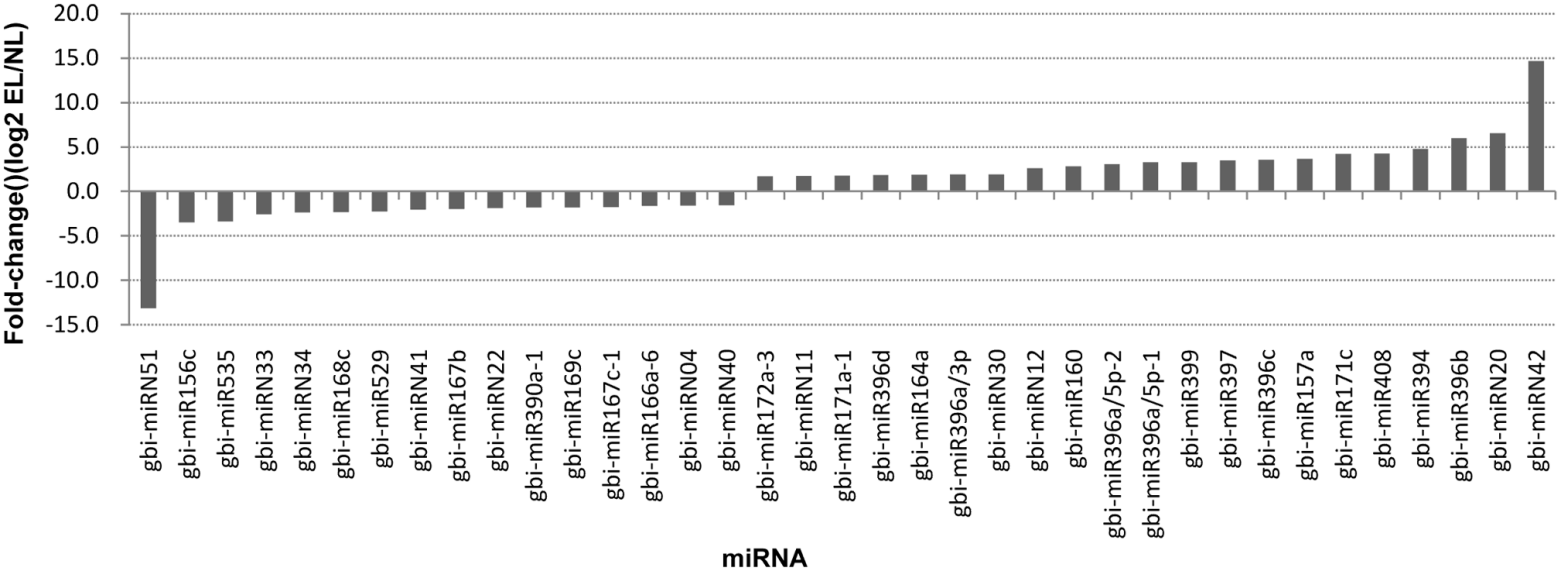
Differentially expressed miRNAs in epiphyllous ovule leaves (EL) compared with normal leaves (NL). Only miRNAs that identified in both datasets with fold change higher than 1.5 are shown.

We identified 82 conserved miRNAs (belonging to 57 members) and 53 novel candidate miRNAs from the *G*. *biloba* var. *epiphylla* EL and NL datasets. The most abundant miRNAs in both datasets were miR156, miR166, miR168, miR172, miR390 and miR396. Some miRNAs, gbi-miR166a-1, gbi-miR166b-1, gbi-miR168a-1 and gbi-miR172a-1 were sequenced more than 10,000 times, while the least abundance miRNAs were from six miRNA families and were less than 100 times. Different members of some families displayed diverse expression patterns; for example, in the miR166 family the abundances ranged from 16 to 385,231 in EL dataset and from 18 to 738,868 in NL dataset (S1 Table). The normalized expression levels of the conserved miRNAs between the two datasets were analyzed further. Among the 82 conserved miRNAs, 25 miRNAs from 16 families were found to be differentially expressed (fold changes >1.5, P-values ≤0.01) between the two datasets. Sixteen of the 25 differentially expressed miRNAs were more highly expressed in EL than in NL (Fig 4). The expression level of gbi-miR396b showed a 6-fold increase in EL compared with N, while the expression level of gbi-miR156c showed a 3-fold decrease (Fig 4).

Thirty-four of the novel candidate miRNAs were expressed in both databases, 11 were specifically expressed in EL and 8 were detected only in NL. The most abundant novel miRNAs were miRN17, miRN43, miRN41 and miRN15. Most of the novel miRNAs were sequenced less than 100 times. The expression patterns of most of the candidate miRNAs identified in both databases were similar. Compared with the miRNA expression levels in NL, 10 were significantly up-regulated and 11 were significantly down-regulated in EL (S2 Table). Among the up-regulated miRNAs, miRN42 had the highest fold change (14.67-fold), while among the down-regulated miRNAs, miRN51 had the highest fold change (13.15-fold). The differentially expressed up-/down regulated miRNAs had fold changes that ranged from 2- to 14-fold.

### Target prediction of *G*. *biloba* var. *epiphylla* miRNAs

To determine the functions of the identified miRNAs, putative target genes were predicted using psRNA Target [38]. A total of 140 *G*. *biloba* var.*epiphylla* unigenes were predicted as possible targets for the 82 mature conserved miRNA sequences (S3 Table). The targets were analyzed using BLASTX searches against Nr database to evaluate the putative functions. More than half of the target genes were annotated as unknown or hypothetical proteins. Many of the annotated target genes were conserved transcription factors, including SQUAMOSA Promoter Binding Protein (SBP) for gbi-miR156, homeodomain-leucine zipper (HD-ZIPIII) for gbi-miR166, auxin response factor (ARF) for gbi-miR160, MYB for gbi-miR159, APETALA2-like for gbi-miR172 and growth-regulating factor for gbi-miR396. The miR171 targeted Scarecrow-like (SCL) transcription factors has been associated with RNA silencing [39]. Besides the conserved transcription factors, some functional proteins may also affect the epiphyllous ovule formation; for example, leucine-rich repeat receptor protein kinase, a target for gbi-miR390. Leucine-rich proteins have been found to be necessary for male gametophyte development and ovule specification and function [39, 40]. The Dicer-like protein (DCL1), an essential component in miRNA biogenesis, was predicted to be target of gbi-miR162. Other predicted target genes include genes that encode laccase, defensin precursor, blue copper protein and ATP binding protein, suggesting that *G*. *biloba* var. *epiphylla* miRNAs may be involved in a wide range of physiological functions.

Additionally, 199 possible target genes were predicted for 49 novel miRNAs (S3 Table) and of these, 136 target genes were annotated as unknown or hypothetical proteins. Most of the novel miRNAs had multiple possible target genes, although for several miRNAs only one target gene was predicted. For example, gbi-miRN06 and gbi-miRN41 were both predicted to target SBP. The functional categories of the annotated target genes were diverse and included hormone regulation (auxin-responsive (IAA) and ARF for gbi-miRN41 and gbi-miRN45, respectively), ligase activity (E3 ubiquitin-protein ligase for gbi-miRN18), various kinases (receptor-like protein kinase, serine-threonine protein kinase) and enzymes involved in metabolic pathways (laccase/ levopimaradiene synthase).

### Annotation of potential targets of differentially expressed miRNAs

**Fig 5:**
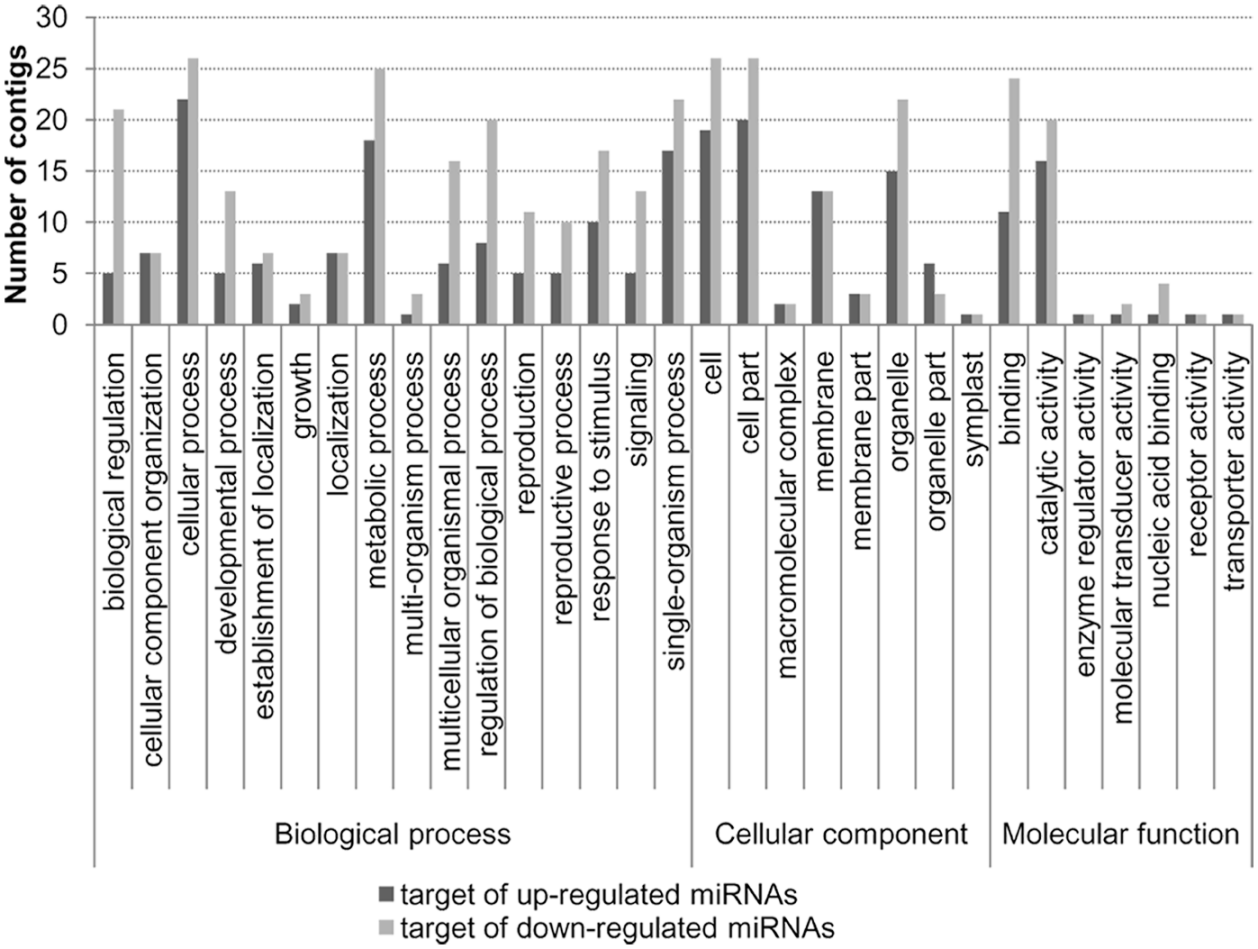
GO annotation of the targets genes of the differentially expressed miRNAs.

To evaluate the potential functions of targets of the differentially expressed miRNAs, gene ontology (GO) annotations were assigned to the targets using Blast2GO software [41]. A total of 111 and 88 unigenes were identified as potential targets of 38 up-regulated and 34 down-regulated miRNAs, respectively, in EL. Categorization of the target genes showed that the genes annotated under biological process fell in to 16 categories of which the three most over-represented GO terms were cellular process, metabolic process, and single-organism process (Fig 5). Eight categories under cellular component and six categories under molecular function were also assigned to the target genes. The functions of the genes targeted by the up-regulated miRNAs in EL were more abundant in biological regulation, development process, reproduction, and signalling process. The results suggested the *G*. *biloba* var. *epiphylla* miRNAs targeted genes participated in a broad range of physiological functions.

### RT-qPCR analysis of selected miRNAs and their predicted targets

**Fig 6:**
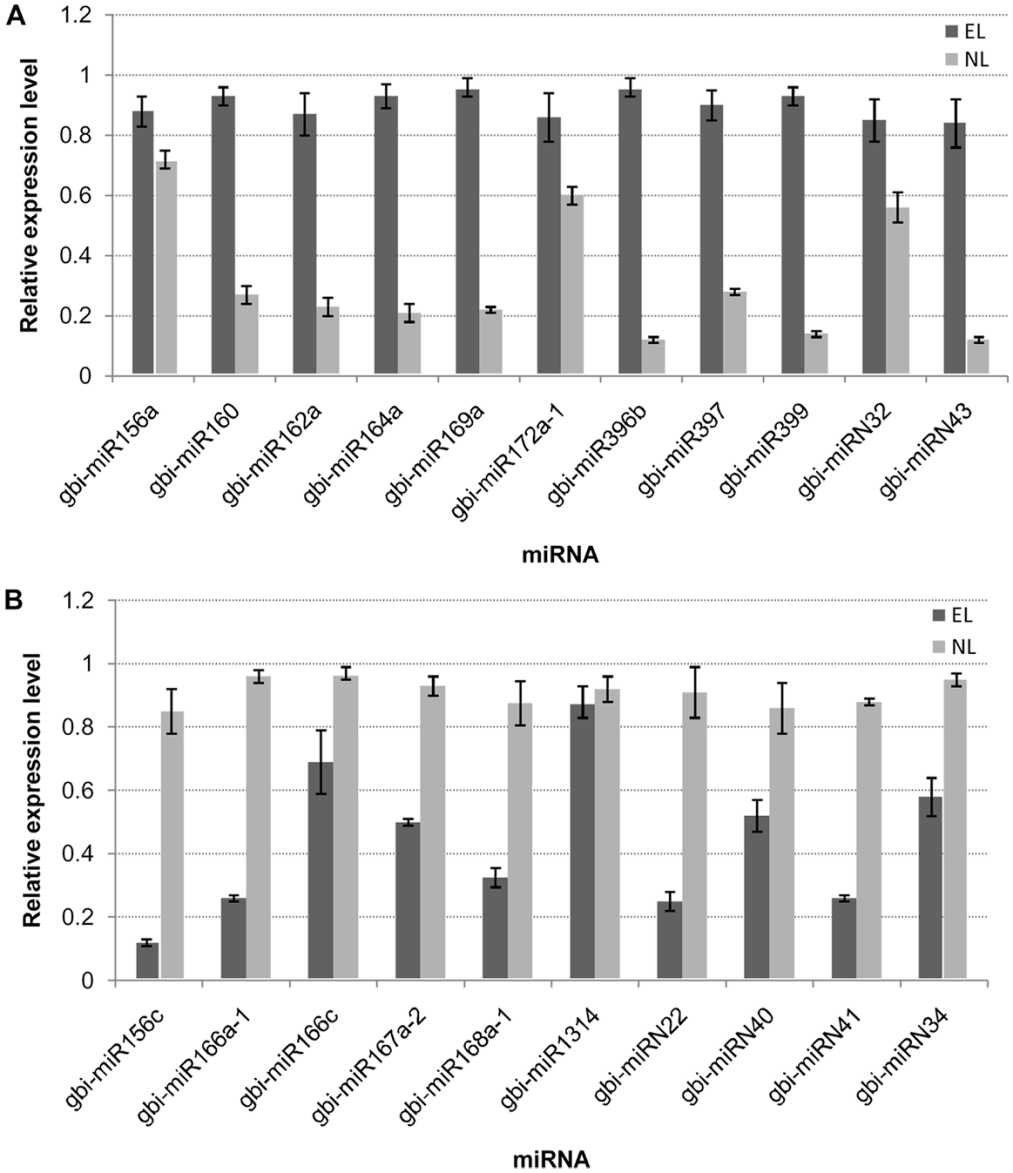
Differentially expressed miRNAs between epiphyllous ovule leaves (EL) and normal leaves (NL) by RT-qPCR. (A) miRNAs with expression levels up-regulated in EL compared with their expression levels in NL. (B) miRNAs with expression levels down-regulated in EL compared with their expression levels in NL. The expression levels of the miRNAs were normalized to that of 18S rRNA. Bars indicated the standard error of means.

**Fig 7:**
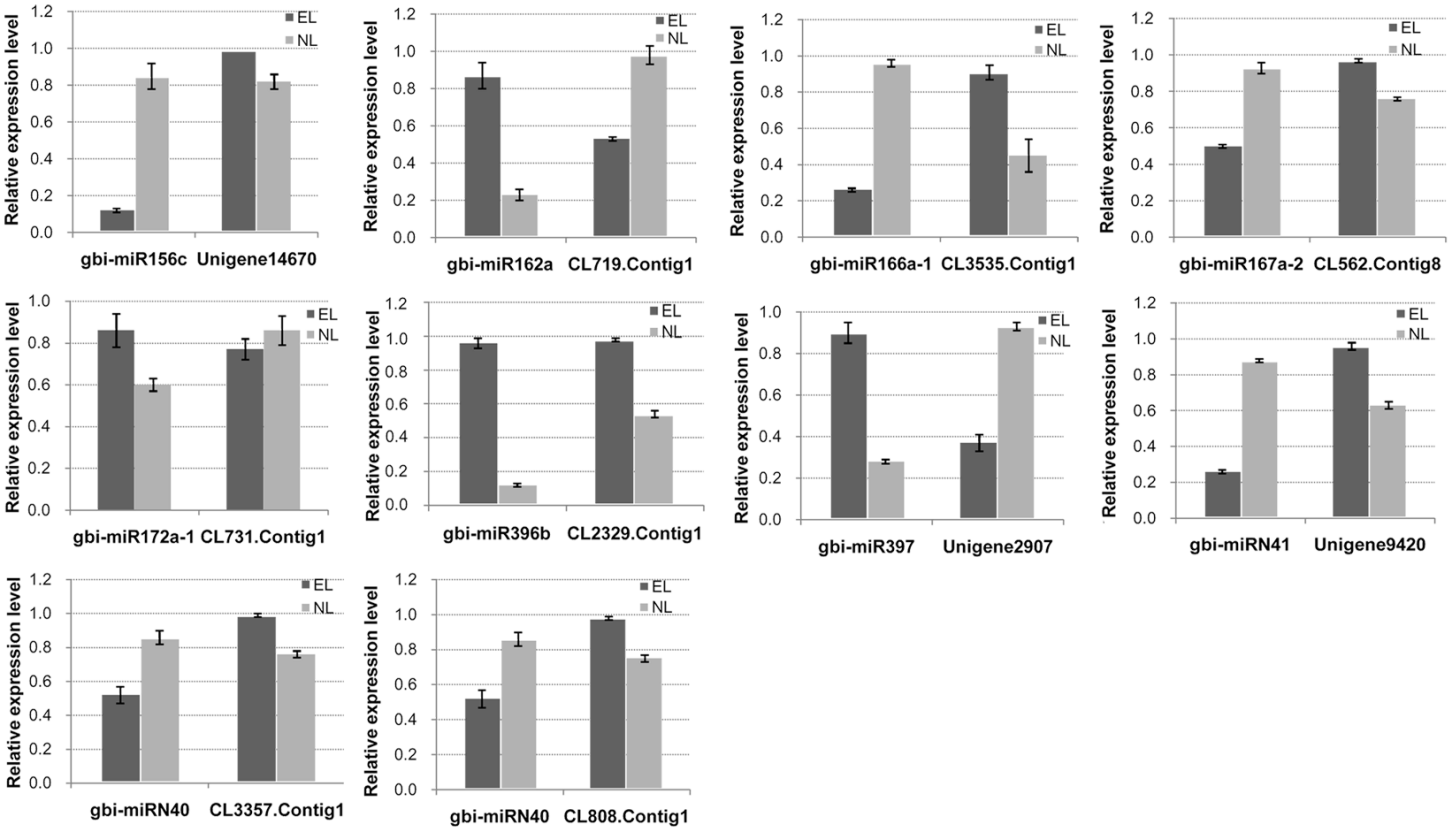
Expression patterns of the ten predicted target genes of the selected miRNAs. The expression levels of the target genes were normalized to that of *GAPDH*. Bars indicated the standard error of means.

To validate the conserved and novel miRNAs obtained from the Solexa sequencing, RT-qPCR was performed on 21 selected miRNAs, including 15 conserved miRNAs and six novel miRNAs. In general, all the miRNA expression profiles were similar to those detected by the Solexa deep sequencing (Fig 6). The expression profiles of the target genes of 9 of the selected miRNAs were also analyzed by RT-qPCR (Fig 7). Except for gbi-miR396b and its target gene (CL2329.Contig1), the expression profiles of the other putative target genes were opposite to those of their corresponding miRNAs. Some selected miRNAs were reported to be associated with the ovule development [23, 42–44]. The RT-qPCR results confirmed the down-regulation of gbi-miR156c and up-regulation of gbi-miR172a-1 in EL. Their target genes Unigene14670 and CL731.Contig1 were highly homologous to SBP and APETALA2 (AP2), respectively. The miR172-targeted AP2/AP2-like transcription factors combined with the miR156-targeted SBP control the juvenile-to-adult transition and the transition to flowering. The down-regulated highly conserved miR166 in EL was validated, and the expression levels of its putative target CL3535.Contig1, which encodes HD-ZIPIII was up-regulated in EL compared with in NL. The RT-qPCR also detected the expression levels of CL562.Contig8, Unigene9420, CL3357.Contig1 which encoded ARF6/ARF8, IAA7 and BEL1-like protein, respectively that were targeted by gbi-miR167a-2, gbi-miRN41 and gbi-miRN40 all of which were down-regulated in EL. Conversely, the CL2329.Contig1, which encodes growth-regulating factor and gbi-miR396b, which targets it, were both up-regulated in EL compared with in NL, implying a complex regulation pattern (Fig 7).

## Discussion

High-throughput sequencing of sRNA transcriptomes from different plant tissues has been used to identify conserved and novel miRNAs and to determine the expression profile of miRNAs. Many angiosperm miRNAs have been identified [8–10, 45, 46], but only a few miRNAs have been reported in gymnosperms [13, 21, 27, 47]. *G*. *biloba* var. *epiphylla* with its unusual epiphyllous ovules is an ideal model plant in which to study the progressive metamorphosis and the ovule formation mechanism in Ginkgo species. We constructed two sRNA libraries of *G*. *biloba* var. *epiphylla* during partly epiphyllous ovule initiation from epiphyllous ovule leaves and from normal leaves. The distribution patterns of sRNAs in *G*. *biloba* var. *epiphylla* revealed significant amounts of 21 nt long RNAs, whereas many studies have reported the distribution patterns in angiosperms have a major peak at 24 nt [30–33, 35]. The length distribution patterns of sRNAs may be related to evolutionary relationships among plant species. The draft assembly genome (20-gigabases) of the first gymnosperm Norway spruce (*Picea abies)* has become available recently [48]. Although the *P*. *abies* genome is 100 times larger than that of *A*. *thaliana*, the number of well-supported genes is similar in both. The accumulation of diverse transposable elements among conifers may have contributed to the large genome size. In conifers, the expression levels of 24 nt sRNAs, which has been implicated in transposable element silencing [49], were found to be much lower than that in other plants [48]. The low expression of 24 nt sRNAs may have contributed to the large genome evolution mechanism in most gymnosperms.

We identified 82 miRNA sequences belonging to 23 conserved miRNA families in the EL and NL datasets. Most of the identified miRNA families were highly conserved in angiosperms and gymnosperms. Some miRNA families, including the miR167, miR168, miR394 and miR399 families were conserved between monocots and dicots, but were not found in gymnosperms. Four miRNA families (miR3522, miR1507, miR1509, miR1314) have been found only in a few species; miR1314 has been found only in gymnosperms [13, 27]. Because the genome sequences information is currently unavailable, pre-miRNAs with characteristic secondary structures could only be predicted for a limited number of miRNAs (S1 Fig). The distribution of conserved miRNAs in two datasets varied widely. The highly conserved miRNAs, such as gbi-miR166a/156a/168a/172a, had higher abundances while the less conserved miRNAs had lower abundances. In addition, 53 novel candidate miRNAs in *G*. *biloba* var. *epiphylla* were identified. For 25 of the novel miRNAs, miRNA*s were also identified. Recent studies have speculated the high accumulated miRNA*s may exert certain functions as miRNAs [7]. We found significant differences in the regulation patterns of both the conserved and novel miRNAs in the two databases. Forty-six miRNAs were found to be differentially expressed in the EL and NL tissues, including some miRNAs that were tissue specific. Target prediction showed that some of the differentially expressed miRNAs may regulate epiphyllous ovule formation.

The targets were annotated as being involved in many biological metabolic processes, such as hormones, substance/energy metabolism, and signal transduction. The phytohormone auxin plays crucial roles in mediating pattern formation during ovule development [23, 50] and in Ginkgo, the concentration of IAA has been reported to increase during the period of ovule rapid growth [51]. ARFs have been found to mediate gene expression responses to auxin by binding to the promoter regions of these genes [50, 52] and the posttranscriptional regulation of ARF6 and ARF8 transcript abundances by miR167 has been reported to be highly conserved in seed plants [8, 47, 53], which is vital for ovule development [42, 54]. Overexpression of miR167 phenocopies of the *arf6/arf8* double mutant, was found to cause arrested ovule integuments growth [55]. Besides, ARF6 and ARF8 may activate auxin response genes in reproductive organs [54]. In the present study, the CL562.Contig8, which is highly homologous to Ginkgo ARF6/8 was predicted as the target gene of gbi-miR167 and Unigene9420, which encodes IAA7, was the predicted target for gbi-miRN41. The expression of both gbi-miR167 and gbi-miRN41 were significantly decreased in EL, and the two auxin response genes they were predicted to target were down-regulated, which may allow the increased accumulation of ARF6/8 and IAA7 in the key stage of epiphyllous ovule germination, indicating the modulation of auxin-dependent patterning.

Thus, many conserved miRNAs in the two libraries targeted genes that may be involved in ovule development. The target genes that encode transcription factors, including miR156-targeted SBP, miR159-targeted MYB, miR166-targeted HD-ZIPIII, and miR172-targeted AP2 may also be important regulators in the key stage of epiphyllous ovule germination. The HD-ZIPIII genes targeted by gbi-miR166 are highly conserved in all lineages of land plants and have been reported to be involved in leaf morphogenesis, ovule development, meristem formation, and vascular development [43, 56]. Previous studies have found that miR166-mediated regulation of the expression of HD-ZIPIII transcription factors (PHB and PHV) may be required for normal ovule development in Arabidopsis. Loss of PHB, PHV, and Ca-2+ dependent nuclease (CAN) was found to lead to arrested or abnormal inner and outer integuments [44, 56]. It has also been reported that HD-ZIPIII and KAN (transcription repressor) activity via PIN1 (auxin efflux carrier) localization may mediate auxin signaling during ovule development and embryogenesis [57]. Besides, a BEL1-like protein (CL3357.contig1) was predicted as the target gene of gbi-miRN40. BEL1, a homeodomain transcription factor, is an important regulator in controlling the integument morphogenesis and development [58]. The *bel1* mutant inhibits the development of the integument, and even affects female gametophyte development [59, 60]. In addition, BEL1 together with SPL/NZZ is also important for auxin/cytokinin signaling during ovule development in Arabidopsis [58]. In the present study, almost all members of the miR166 family showed lower expression levels in the EL tissues compared with the NL tissues, but differences were not significant. In addition, gbi-miRN40, which also targeted BEL1, was down-regulated by 1.5 folds in the EL tissues. The validated up-regulated HD-ZIP III and BEL1-like encoding genes in epiphyllous ovule leaves may be involved in epiphyllous ovule germination and the auxin signaling transduction pathway.

The highly conserved miR156/SPL interaction model has been found in evolutionarily distant species, including liverwort, angiosperms, and gymnosperms [11, 14, 61]. SQUAMOSA Promoter-Binding Protein-Like (SPL) genes play crucial roles in plant developmental phase transition, floral meristem identity, leaf morphology, and sporogenesis initiation [62–64]. MiR156 regulates both juvenile-to-adult phase transition and vegetative-to-reproductive phase transition by repressing the expression of SPLs. A sufficient amount of miR156 is necessary to promote the juvenile phase; for example, overexpression of miR156 in Arabidopsis was reported to prolong the juvenile phase and even delay flowering time [65–67], while the overexpression of the miR156-resistant SPL transgenes (rSPL3, rSPL4, rSPL5) was found to result in early flowering [65, 68, 69]. The miR172-targeted AP2/AP2-like transcription factors were also reported to control juvenile-to-adult transition and the transition to flowering [70, 71]. Thus, miR172 and miR156 seem to regulate transitions that oppose each other, perhaps because miR156-targeted SPLs directly regulate the AP2-like floral repressors and are indirectly regulated by binding to miR172 [65, 72]. Compared with NL, gbi-miR156c was significantly reduced in EL, and miR172 a/b were found to be up-regulated. The expression levels of these miRNAs and the opposite expression patterns of the target SBP genes (Unigene14670) and AP2 (CL731. Contig1) were validated by RT-qPCR. Similar to the juvenile-to-adult/ vegetative-to-reproductive phase transition, the sequential activity of miR156c and miR172 may be involved in the normal to epiphyllous ovule leaves transition.

## Materials and Methods

### Ethics Statement

The Plant materials were used from 1300 year-old *Ginkgo biloba* var. *epiphylla* in Yiyuan County, Shandong Province, China (36°11′N, 118°09′E). We got the permission of collecting the plant materials from the Yiyuan County Forestry Bureau. No specific permissions were required for these activities. And the field studies did not involve endangered or protected species.

### Sample preparation and RNA extraction

The Plant materials were used from 1300 year-old *Ginkgo biloba* var. *epiphylla* in Yiyuan County, Shandong Province, China (36°11′N, 118°09′E) with the permit of the Yiyuan County Forestry Bureau. Epiphyllous ovule leaves (EL) and normal leaves (NL) were collected in the early stage of the epiphyllous ovule germination. The leaves were immediately frozen in liquid nitrogen and then transferred to -80°C freezer until used. Because of the large amounts of flavonoid glycosides, terpene lactones, carbohydrate polymers, phenolic compounds, and many other secondary metabolites in Ginkgo leaf, an improved CTAB protocol [73] was developed to isolate total RNA with high quality and integrity. Two single RNA pools from EL and NL were formed.

### Small RNA sequencing library construction

The total RNA was isolated from EL and NL using the improved CTAB protocol. After removing the residual DNA with RNase-free DNase I, the RNA was separated on 15% denaturing PAGE for size selection. The sRNA of 18–30 nt were ligated sequentially to 3’ and 5’ adaptor using T4 RNA ligase and amplified by reverse-transcription PCR (RT-PCR). The purified PCR products were isolated from agarose gel and sequenced on an Illumina/Solexa Sequencer according to the manufacturer's instructions at the Beijing Genomics Institute, China.

### Identification of conserved and novel miRNAs

The raw sequence reads obtained by Solexa sequencing were filtered to remove low quality reads and contaminants including products of multiple adapter ligation and empty constructs. The remaining clean sequences were used for further analysis (Deep-sequencing data have been deposited to the Sequence Read Archive at the NCBI database with accession number SRP050106). Firstly, rRNA, tRNA, snRNA, and snoRNA sequences were removed by comparing the read sequences against non-coding sRNA sequences deposited in the NCBI Genbank database and Rfam database (http://rfam.sanger.ac.uk). Then, the unique sRNA sequences that sequenced at least ten reads in both the libraries were used in BLASTN searches against all non-redundant plant mature miRNAs that were deposited in the miRNA database (miRBase 20.0) [74], to identify conserved miRNAs in the *Ginkgo biloba* var. *epiphylla* EL and NL libraries. Only perfectly matched sequences were considered to be conserved miRNAs. Then the unannotated sRNAs were used to discover potential novel miRNAs. To further obtain the conserved and novel miRNA precursors, the unique sRNAs were aligned to the *G*. *biloba* mRNA transcriptome sequences using the MIREAP (http://sourceforge.net/projects/mireap/). Then the stem-loop structures of the pre-miRNAs were folded using Mfold [75]. These precursor sequences were retrieved and used for BLASTX analysis to remove the possible coding sequences. By further BLASTN analysis, the remaining putative miRNAs with no homolog matching with the known miRNAs were considered as novel miRNAs. Finally, the criteria proposed by Meyers *et al*. [76] was used to annotate the candidate miRNAs.

### Differential expression analysis of miRNAs

To compare the differential miRNA expression between the EL and NL libraries, fold change and P-value were considered together as following: first, the expression of the miRNAs in EL and NL were normalized to reads per million; next, fold change and P-value were calculated between the two libraries; then the fold changes and P-values were considered to determine the significantly differentially expressed miRNAs between the two samples. If the fold change (log2) was >1.5 or <−1.5 and the P-value was ≤0.01, then it was considered to indicate an extremely significant difference in expression levels. When the fold change was the same as the one above, but the P-value was >0.01 but <0.05 it was considered to indicate a significant difference in expression levels; otherwise no significant difference between the two samples was recorded. If the fold changes in the two libraries were both <1, in the corresponding reads were not included in the analysis. The following formulas were used to calculate (1) normalized expression = (number of actual miRNA reads / total number of clean reads) ×1,000,000; (2) fold change = Log2 (EL normalized expression level/ NL normalized expression level); and (3) P-value:

$$p\left(x|y\right)=\left(\frac{N_{2}}{N_{1}}\right){\frac{\left(x+y\right)!}{x!\left(y\right)!{\left(1+\frac{N_{2}}{N_{1}}\right)}^{\left(x+y+1\right)}}}_{D\left(y\geq y_{\max}|x\right)=\sum_{y\geq y_{\max}}^{\infty }p\left(y|x\right)}^{C\left(y\leq y_{\min}|x\right)=\sum_{y=0}^{y\leq y_{\min}}p\left(y|x\right)}$$

*N1* and *x* represent total number of clean reads of one miRNA family and normalized expression level respectively in EL, and *N2* and *y* represent total number of clean reads of one miRNA family and normalized expression level respectively in NL.

### Prediction of potential target of *G*. *biloba* var. *epiphylla* miRNAs

The newly identified and conserved *G*. *biloba* miRNAs were searched against the *G*. *biloba* tanscriptome mRNA database using the web tool psRNATarget with default parameters [38] to identify potential miRNA target genes. Then, the identified candidate targets were searched against the Nr database using BLASTX with E-values less than e^-10^ to predict their possible functions. The predicted targets of the differential expressed miRNAs were annotated with GO terms using the Blast2GO software [41].

### Quantitative RT-PCR analysis of selected *G*. *biloba* var. *epiphylla* miRNAs

Small RNAs was isolated from the EL and NL tissues using the Plant RNA Isolation Kit (Bioteke, Beijing, China) and purified by RNase-free DNase I treatment (Promega, Madison, USA). Then poly (A) polymerase-mediated RT-qPCR was used to investigate the expression of 21 selected miRNAs. The cDNAs were synthesized from 500 ng of sRNA with the TaKaRa One Step Primescript miRNA cDNA Synthesis Kit (Takara, Dalian, China) following the manufacturer’s recommendations. The 25 μl miRNA reverse transcription reaction mixture was incubated at 37°C for 60 min, followed by 5 s at 85°C. Then SYBR Premix ExTaq II Kit (TaKaRa) was used for the RT-qPCR on a CFX96 Detection System (Bio-Rad, Hercules, California, USA). The reactions were incubated in a 96-well plate at 95°C for 10 s, followed by 45 cycles of 95°C for 5 s and 65°C for 30 s. The melt curve was chosen as the default. The snRNA 18S (GenBank Accession No. D16448.1) was used as an endogenous control. The 2^-ΔΔCT^ method was used to analysis the relative expression levels. The RT-qPCR was performed using three biological replicates for credible statistical analysis. The miRNA-specific primer sequences and the primers for the reference 18S gene are shown in S4 Table.

### Validation of predicted target genes by RT-qPCR

Total RNA from EL and NL tissues was isolated using the improved CTAB protocol [73]. The purified RNA was reverse-transcribed using a RevertAid First Strand cDNA Synthesis Kit (Thermo, EU) according to manufacturer’s protocol. Specific primers for 10 target genes were designed using Primer Premier 5.0 (S4 Table). The RT-qPCR was performed using the SYBR Green PCR Master Mix (TaKaRa) on CFX96 Detection System (Bio-Rad). Briefly, the 25 μl PCR reaction contained no more than 100 ng cDNA, 12.5 μl SYBR Premix DimerEraser (2×) and 0.3 μM each primer. The reactions were mixed and incubated at 95°C for 30 s, followed by 40 cycles of 95°C for 5 s, 55°C for 30 s and 72°C for 30 s. The expression levels were normalized to the internal control GAPDH [77, 78]. The relative expression levels were also analyzed using the 2^-ΔΔCT^ method. Three biological replicates were performed for each sample.

## Supporting Information

S1 Fig Secondary structures of the conserved miRNA precursors from *G*. *biloba* var. epiphylla. (TIF)

S1 Table Conserved miRNAs and their differential expression patterns in the epiphyllous ovule leaves and normal leaves libraries. (XLSX)

S2 Table Precursors and primary sequences of novel miRNAs in *G*. *biloba* var. *epiphylla*. (XLSX)

S3 Table Predicted targets of conserved and novel miRNAs. (XLSX)

S4 Table Primers for the RT-qPCR of miRNAs and their putative target genes. (XLSX)
