# Supplementary figures and images for: Identification and Characterization of MicroRNAs in Ginkgo biloba var. epiphylla Mak
Source: PLoS One. 2015 May 15;10(5):e0127184. doi: 10.1371/journal.pone.0127184 (PMC4433266; doi:10.1371/journal.pone.0127184)

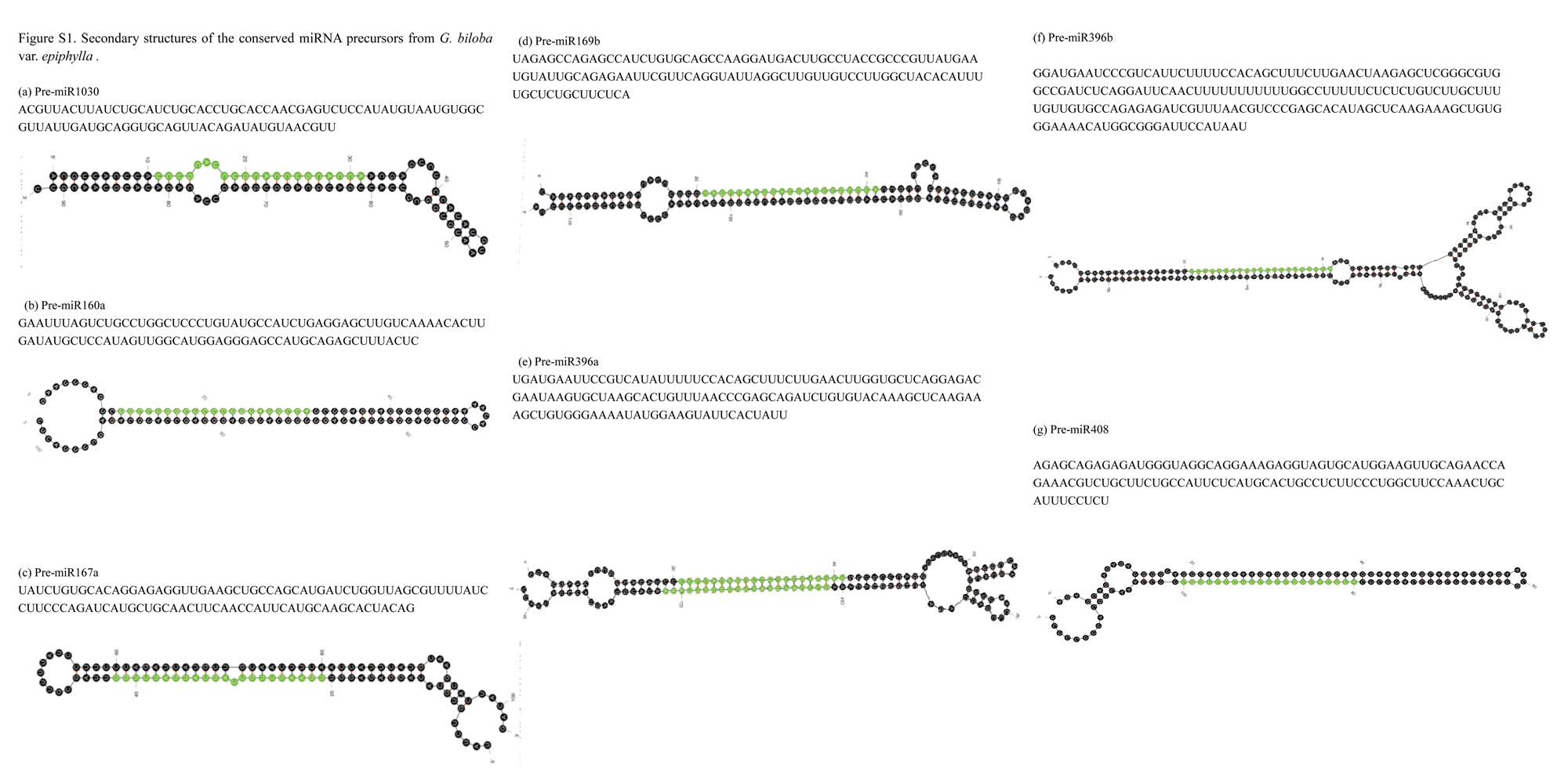

Supplement: S1 Fig — (TIF) [file pone.0127184.s001.tif]
